# A systematic review of machine learning models for management, prediction and classification of ARDS

**DOI:** 10.1186/s12931-024-02834-x

**Published:** 2024-06-04

**Authors:** Tu K. Tran, Minh C. Tran, Arun Joseph, Phi A. Phan, Vicente Grau, Andrew D. Farmery

**Affiliations:** 1 Nuffield Division of Anaesthetics, University of Oxford, Oxford, UK; 2 Department of Engineering and Science, University of Oxford, Oxford, UK; 3 Nuffield Department of Clinical Neurosciences, Oxford Institute of Biomedical Engineering, University of Oxford, Oxford, UK

**Keywords:** AI, ARDS, Explainable AI

## Abstract

**Aim:**

Acute respiratory distress syndrome or ARDS is an acute, severe form of respiratory failure characterised by poor oxygenation and bilateral pulmonary infiltrates. Advancements in signal processing and machine learning have led to promising solutions for classification, event detection and predictive models in the management of ARDS.

**Method:**

In this review, we provide systematic description of different studies in the application of Machine Learning (ML) and artificial intelligence for management, prediction, and classification of ARDS. We searched the following databases: Google Scholar, PubMed, and EBSCO from 2009 to 2023. A total of 243 studies was screened, in which, 52 studies were included for review and analysis. We integrated knowledge of previous work providing the state of art and overview of explainable decision models in machine learning and have identified areas for future research.

**Results:**

Gradient boosting is the most common and successful method utilised in 12 (23.1%) of the studies. Due to limitation of data size available, neural network and its variation is used by only 8 (15.4%) studies. Whilst all studies used cross validating technique or separated database for validation, only 1 study validated the model with clinician input. Explainability methods were presented in 15 (28.8%) of studies with the most common method is feature importance which used 14 times.

**Conclusion:**

For databases of 5000 or fewer samples, extreme gradient boosting has the highest probability of success. A large, multi-region, multi centre database is required to reduce bias and take advantage of neural network method. A framework for validating with and explaining ML model to clinicians involved in the management of ARDS would be very helpful for development and deployment of the ML model.

## Introduction

Acute respiratory distress syndrome (ARDS) is a common complication in adult general intensive care units (ICUs) [1]. In 2016 a survey conducted in 459 ICUs across 50 countries demonstrated that ARDS occurred in 10% of patients with a mortality rate exceeding 40% [1]. The management of ARDS in the US, UK and Europe is largely based on the individual country’s national guidelines. Although these guidelines are created based on nationwide surveys and research studies, the quality of evidence for recommendations for clinical practice is poor with absence of high-quality evidence [2]. This may explain why there is a poor uptake of the guidelines by clinicians. For example, the UK guidelines recommend a low tidal volume of less than 8ml/kg and a positive-end expository pressure (PEEP) of more than 12 cmH_2_O [2]. However, only about 60% of patients received 8ml/kg of tidal volume or less and more than 82% received less than 12cmH_2_O PEEP [1]. Huge practice variations are recognised and there is an urgent need for evidence-based and standardised management for ARDS in ICU.

Machine learning (ML) has been applied successfully into other areas including natural language processing, computer vision applications, and automatic speech recognition. As a result, advancement has been made in many areas from sports to robotic, from entertainment to industry. Applications of ML have shown enormous potential across several medical fields such as disease prediction, clinical outcome prediction, diagnosis and prognosis using various data modalities, including time signals and medical imaging [3–18].

Although ML has the ability to recognise patterns within large amount of data, many of these patterns are imperceptible by human. These patterns can be used in different ways to categorise or predict events [3]. However, to be successfully integrated into the health care system, ML applications must aim to archive high performance metric such as accuracy and achieve trust from users towards clinical application. As a result, the demand for better transparency in ML models in medicine is essential for better understanding of the causality and relationship between input and output, and for legal and ethical purposes [19–21].

The concept of interpretation or explainability in machine learning is defined as the capability of the algorithm to present and/or produce knowledge contained inside the data so that it is perceptible and understandable by users [22]. Various explainability methods have been used in medical care in general [23] and for ARDS data in particular [24]. However, few studies have actually validated the effectiveness of these explainability methods with direct involvement of clinicians [23]. There is also lack of evidence on which method is most suitable for clinicians in terms of its explainability.

The main focus of this review is to identify studies that has used machine learning methods on the management, prognosis and diagnosis of patients with ARDS, reflect on usage of different database and data gathering method, algorithms and their effectiveness. The review also aims to highlight the state of explainability in term of methods and usages, and performance of different ML methods in ARDS.

## Method

### Inclusion and exclusion criteria

Articles employing machine learning or artificial intelligence addressed directly to the diagnosis, management, risk assessment, prognosis or outcome of ARDS were included in the review. The included articles can utilise existing ML algorithm or create new algorithm based on either classical ML method such as decision tree or more advanced ones like neural network or both. Protocol, commentaries, letters, abstract-only articles, conference proceedings, non-English and non-peer reviewed articles were excluded. Only studies using exclusively human data were selected. Research using paediatric patients was excluded.

### Search strategy

**Fig. 1:**
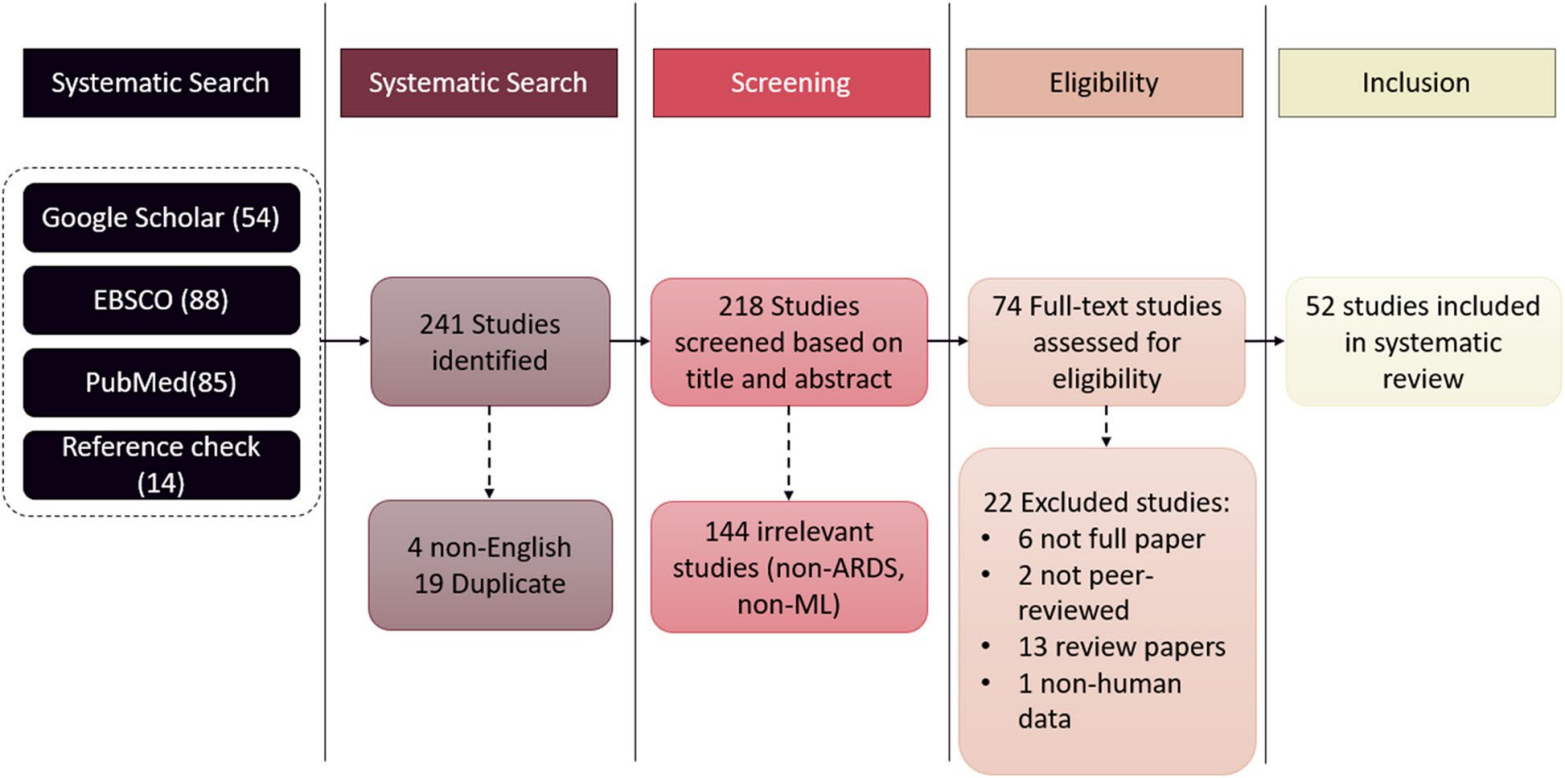
The PRISMA diagram for this review. The authors checked all records for eligibility. In a total of 243 studies identified from Google Scholar, EBSCO, PubMed and reference screening, 52 studies were included in this review

An extensive literature search was performed in Pubmed, Google scholar and EBSCO on July 2023. The summary of the screening process is reported in the PRISMA diagram (Fig. 1) A random snowball search was also carried out using Google to identify any additional results. Keywords used for these searches include “acute respiratory distress syndrome”,”ARDS”, “acute lung injury”, “ALI”,”machine learning” and”artificial intelligence”. Boolean Operator “AND” and “OR” was used for Pubmed and EBSCO searches. The reference list of all results was also screened by title and abstract for potentially relevant citations. The list of author contributions to this paper is included.

All the search results were collected using their title and abstract. The full-text version of these results was used for screening using criteria in 2.1. Non-full-text paper was excluded at this stage. This process was carried out independently by TT and MT to eliminate bias and disagreements were resolved with consensus from all authors.

## Results

### Search results and selection process

Google Scholar search yielded 54 results after preliminary screening. Three non-English articles were excluded along with 2 not yet peer-reviewed results, 1 duplication and 27 irrelevant articles. One duplicated paper was also excluded.

The search was repeated with the EBSCO and Pubmed database, resulting in 88 articles and 85 articles respectively. Finally, 52 articles were selected for review matching all criteria listed in Inclusion and exclusion criteria section (Table 1).

**Table 1:** Overview of studies included in this systematic review

Year	Author	Area of research	Database and data source	Method of ML	Performance metric, result and best-performed algorithm	Explainability
2009	Herasevich, et al., [25]	diagnose ARDS	3795 patients from 9 multidisciplinary ICUs	ALI sniffer algorithm	sensitivity = 96%	
2009	Pearl, et al., [26]	predict ARDS	NTDB: 1,438,035 patients	NN	accuracy = 93.8%	feature importance
2011	Brown, et al., [27]	predict mortality	2022 participants from 2002 ARDS network trial	classification tree	AUC = 0.71	
2011	Koenig, et al., [28]	diagnose ARDS	1270 patients from hospital of the University of Pennsylvania	assist acute lung injury selection system to identify subjects for treatment/trial	sensitivity = 97.6%	
2012	Chbat, et al., [29]	predict ARDS	Multidisciplinary Epidemiology and translational research in Intensive Care (METRIC) Datamart: 526 patients	rule-based fuzzy inference system, Bayesian network, finite state machine	sensitivity = 71.7–92.6%	
2013	Bernstein, et al., [30]	ARDS management	Questionnaire: 6 clinicians	fuzzy logic		
2018	Sinha, et al., [31]	ARDS subphenotypes	SAILS database: 745 Patients	latent class analysis	hypo-hyper inflammatory	
2018	Afshar, et al., [32]	diagnose ARDS	533 patients of 8255 radiology report	natural language processing with linear SVM	AUC = 0.81	
2019	Zeiberg, et al., [33]	prediction of ARDS	1621 patients in 2016, 1122 patients in 2017	logistic regression, XGBoost	AUC = .81	feature importances (weight)
2019	Yu, et al., [34]	predict ARDS using lung heart pressure index	MIMIC 3: 448 patients	random forest	AUC = .708	
2019	Zampieri, et al., [35]	predict heterogeneity in treatment in ARDS trial patients	1010 patients enrolled in the ARDS trial	Bayesian regression	classified model -3 group	
2019	Zhang, et al., [36]	predict mortality of ARDS	ARDSnet: 5159 patients	tree-based gradient boosting	AUC = 0.748	
2019	Ding, et al., [37]	predict ARDS	296 patients in China	random forest	AUC = 0.82	
2019	Zhang, et al., [38]	predict the mortality of ARDS patients	1071 patients from 44 hospitals	NN	AUC = 0.821	feature importance
2019	Zhou, et al., [39]	predict ARDS	48 patients with 85 breaths samples	principal component analysis, linear discriminant analysis	accuracy = 87.1%	
2020	Yang, et al., [40]	diagnose ARDS	MIMIC 3: 8702 patients	logistic regression, adaboost, XGBoost, NN	AUC = 0.9128 XGBoost	
2020	Reamaroon, et al., [41]	diagnose ARDS	401 patients between February and March 2016	SVM	AUC = 0.8548	
2020	Sinha, et al., [42]	ARDS subphenotype	ARMA + ALVEOLI = 2022 patients, validate by SAILS = 745 patients	gradient boosting (XGBoost)	AUC = 0.95	
2020	Le, et al., [43]	early ARDS prediction	MIMIC 3: 9919 patients	XGBoost	AUC = 0.905	
2020	Hu, et al., [44]	predict mortality of ARDS patients	217 patients in China	NN, logistic regression	AUC = 0.854	feature importances
2020	Chen, et al., [45]	diagnose ARDS via CT for covid patients	86 patients with 352 CT scan images	logistic regression + linear regression	AUC = 0.91	
2020	Sinha, et al., [46]	ARDS subphenotype	ARMA, ALVEOLI, FACTT: 2022 patients total	random forest, bootstrapped aggregating, least shrinkage and selection operator, logistic regression	best AUC = 0.94 logistic regression	feature importances by random forest, bootstrapped aggregating, least shrinkage and selection operator to select the top 6 most important features for the logistic regression model
2021	Xu, et al., [47]	predict ARDS on COVID-19 patients	659 COVID-19 patients in Wuhan	decision tree, logistic regression, random forest, SVM, DNN	best accuracy: tree 98%	
2021	Sayed, et al., [48]	ARDS subphenotype/severity	First 3 ICU days: MIMIC 3 = 2738, 1519, and 1341 patients, eICU = 5153, 2981, and 2326 patients	light gradient boosting, random forest, XGBoost	p/fe ratio better use than pf ratio	
2021	Singhal, et al., [49]	predict ARDS from COVID-19	899 COVID-19 patients from 9 hospital in the US	XGBoost	AUC = .89	SHAP
2021	Sayed, et al., [24]	Mechanical ventilator duration on ARDS patients	MIMIC 3 = 2466 patients, validation = eICU = 5153 patients	light gradient boosting, random forest, XGBoost	light boosting best lowest RMSE Scenario I: 6.08 ± 0.72, Scenario II: 5.87 ± 0.67, Scenario III: 5.93 ± 0.44, Scenario IV: 5.71 ± 0.55	
2021	Sinha, et al., [50]	identify heterogeneity of treatment effect clustering in ARDS patients	ALVEOLI: 549 patients, FACTT: 1000 patients, SAILS: 745 Patients	k-means, partitioning around medoids, hierarchical clustering, spectral clustering, latent class analysis model-based recursive partitioning, casual forest, x-learner with random forest, Bayesian additive regression tree	none of the ML algorithms can consistently identify the cluster	
2021	Schwager, et al., [51]	ARDS subphenotype	eICu = 51,555 patients	multiclass gradient boosting	AUC = 0.77	
2021	Afshin-Pour, et al., [52]	diagnose ARDS	1263 patients from 12 hospitals in New York State	logistic regression, random forest	AUC = 0.85	
2021	Liu, et al., [53]	ARDS subphenotype	eICU = 3875 patients	k-mean	3 subphenotypes	
2021	Lam, et al., [54]	predict ARDS	29,127 patients from 7 US hospitals	RNN	AUC = 0.78	feature inflection map
2021	Huang, et al. [55],	Mortality prediction for ARDS patients	MIMIC 3: 2235 patients, eICU: 331patients	random forest	AUC = 0.905	feature importance
2021	Sabeti, et al., [56]	diagnose ARDS	485 patients from University of Michigan Hospital	SVM-based	best accuracy = 92.62% AUC = 90.52	
2021	Reamaroon, et al., [57]	diagnose ARDS	500 patients with 3078 chest x-rays	SVM, random forest, adaboost, random under-sampling boosting, robust boost, total boost	accuracy = 83% AUC = 79% adaboost	
2022	Lazzarini, et al., [58]	predict the progression of ARDS from COVID-19	289,351 US COVID-19 patients in April 2020	light gradient boosting, random forest, logistic regression	tree and gradient boosting, accuracy = 95%	sharp on gradient boosting
2022	Bai, et al., [59]	predict sepsis-induced ARDS, predict subphenotype	eICU = 5947 patients, MIMIC 4 (validation) = 2699 patients	naïve Bayes, logistic regression, gradient boosted tree, decision tree, random forest	decision tree with AUC = 0.895, 3 phenotypes based on mortality rate	
2022	McKerahan, et al., [60]	predict hypoxic respiratory failure and ARDS	2078 patient from Emory University Healthcare ICUs	XGBoost	AUC = 0.79 for 6h and 0.72 for 24h	
2022	Maddali, et al., [61]	ARDS subphenotype	ARMA + ALVEOLI + FACTT = 2022, testing SAILS = 745, validated: EARLI (*n* = 335) and VALID (*n* = 452)	XGBoost	AUC = 0.92	hypo-hyper inflammatory
2022	Izadi, et al., [62]	prediction of ARDS for COVID-19 patients	8633 COVID-19 patients from 74 countries	k- nearest neighbour, SVM, lasso and elastic-net regularized generalized linear models, generalized additive models, gradient boosting, NN	AUC = .78 gradient boosting	
2022	Jabbour, et al., [63]	diagnose ARDS	Michigan Medicine: 1618 patients, MIMIC IV (validate)	logistic regression + 2 layers NN, CNN + DenseNet-121	AUC = 0.88 combined model	Feature importance
2022	Wu, et al., [64]	prediction of ARDS severity	8 patients from MIMIC 3 and eICU	random forest	AUC = 0.9127	interpretable random forest (Sirus)
2022	Pai, et al., [65]	diagnose ARDS using X-ray	1577 patients in Taiwan	XGBoost, random forest, logistic regression, CNN	best AUC = 0.925 XGB + RF + LR + CNN	SHAP, feature importance
2022	Lam, et al., [66]	predict ARDS	40,703 patients from 7 hospitals	RNN, XGBoost	AUC = 0.842 for RNN	SHAP
2023	Wang, et al., [67]	predict ventilation duration for ARDS patients	MIMIC 4: 1148 patients, eICU-crd: 1697 patients, amsterdamUMCdb: 29 patients	SVM linear kernel, SVM radial basis function kernel, decision tree, random forest, XGBoost, NN, kNN	XGBoost (smallest root mean square error)	SHAP, LIME, DALEX
2023	Zhang, et al., [68]	prediction of ARDS in patients with acute pancreatic	460 patients from China	SVM, ensemble of decision tree, Bayesian classifier, monogram model	AUC = 0.891 Bayesian classifier + ensemble of decision tree best result	
2023	Wu, et al., [69]	predict ARDS and predict severity	eICU: 4738 patients	light gradient boosting, adaboost, random forest, naïve Bayes, logistic regression, SVM, kNN	light gradient boosting best overall, accuracy = 0.911 AUC = 0.8745 for ARDS severe	
2023	Fonck, et al., [70]	detection of ARDS using X-ray	Chexpert: 224,316 images from 65,240 patients, MIMIC-CXR = 377,110 images from 65,379 patients	ResNet-50	AUC = 0.926	
2023	Yahyatabar, et al., [71]	Diagnosis through chest X-ray	373 images from 3 data sources	Dense-Ynet(CNN)	precision = 88.02%	
2023	Barakat, et al., [72]	modelling ARDS patient	1,000,000 simulations based on MIMIC 3 database	NN (fully connected, convolutional layers)	accuracy 90%	
2023	Zhang, et al., [73]	predicts ARDS for patients with severe acute pancreatitis	440 patients from China	logical regression, random forest, SVM, decision tree, XGBoost, NN	AUC = 0.84 best NN	SHAP
2023	Wang, et al., [74]	predicts ARDS in traumatic brain injury patients	MIMIC 3 = 649 patients	XGBoost, light gradient boosting, random forest, adaptive boosting, complement naïve Bayes, SVM	AUC = 1.0 random forest	
2023	Fazaneh, et al., [75]	diagnose ARDS using a chest X-ray	University of Michigan hospital: 414 X-rays from 115 patients	AI aid physician, physician aided AI, average physician and AI, weighted average	physician aided AI, weight average = 0.869 Accuracy	

*AUC* Area under the curve, *SVM* Support vector machine, *NN* Neural network, *DNN* Deep neural network, *RNN* Recurrent neural network, *CNN* Convolutional neural network, *kNN* k nearest neighbour, *XGBoost* Extreme gradient boosting, *MIMIC* The Medical Information Mart for Intensive Care database, *SAILS* Anonymised Data Linking database, *ARMA, ALVEOLI, FACTT* the National Lung, Heart, and Blood Institute ARDS Network database, *SHAP* Shapley additive explanations, *LIME* Local interpretable model-agnostic explanations

### Characteristics of the reviewed studies

**Fig. 2:**
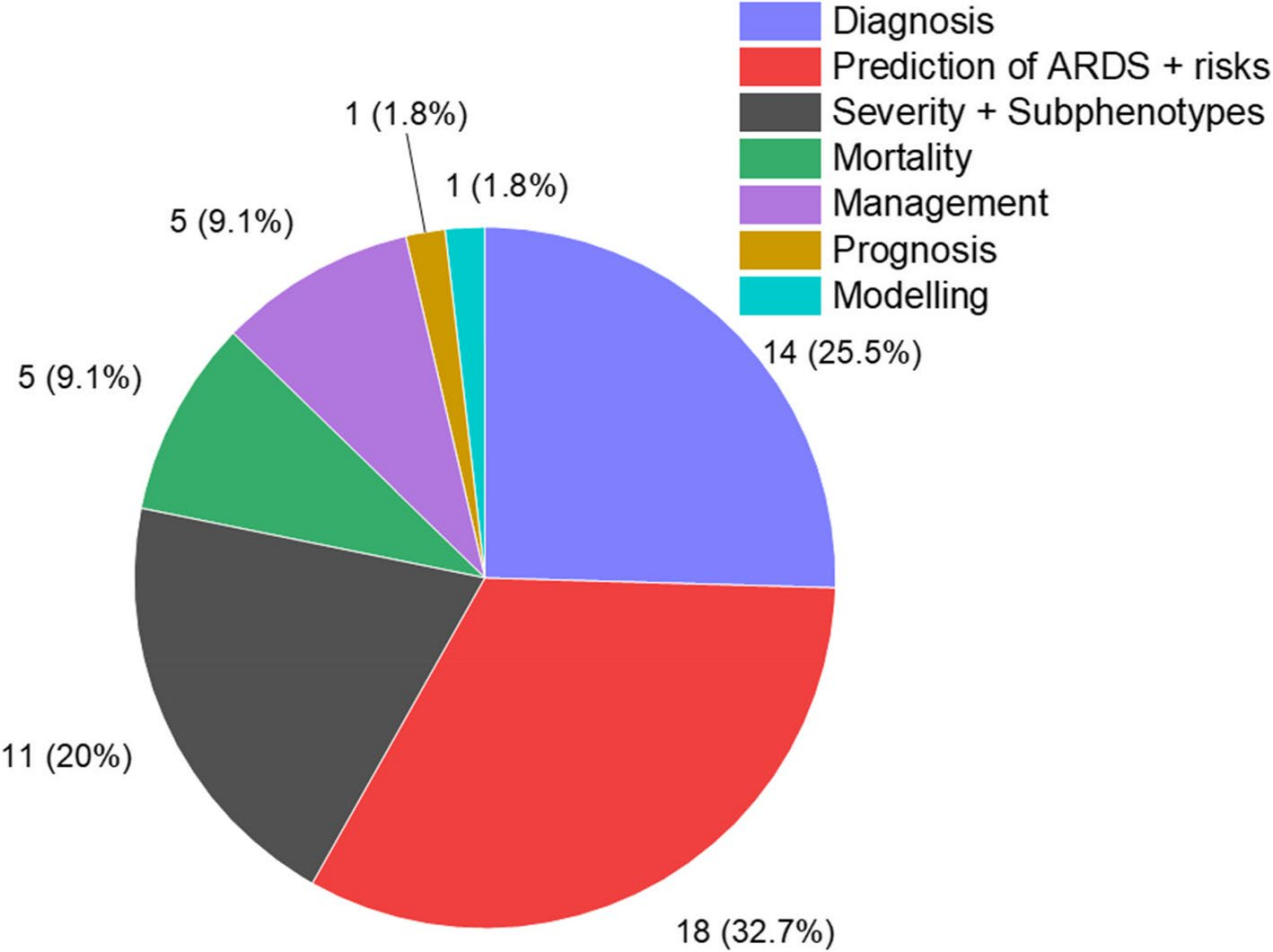
Pie chart of the articles studying the applications of Machine Learning in ARDS. Note that the total number is not 52 because some articles focused on more than one aspect

Fifty-two articles between 2009 and 2023 were selected. 18 (34.6%) of these focused on prediction of ARDS development in patients during hospitalisation. 14 (26.9%) publications articles were related to diagnostic accuracy. 11 (21.2%) articles were focused on categorizing patients with ARDS into groups or subgroups based on severity or mortality. Five articles were related to the use of ML to predict patient mortality or create more suitable management for patients. There is a single (1.9%) article on the prognosis or health trajectory of ARDS and 1 (1.9%) article on using ML to model the condition of patients with ARDS. This can be seen in (Fig. 2).

**Fig. 3:**
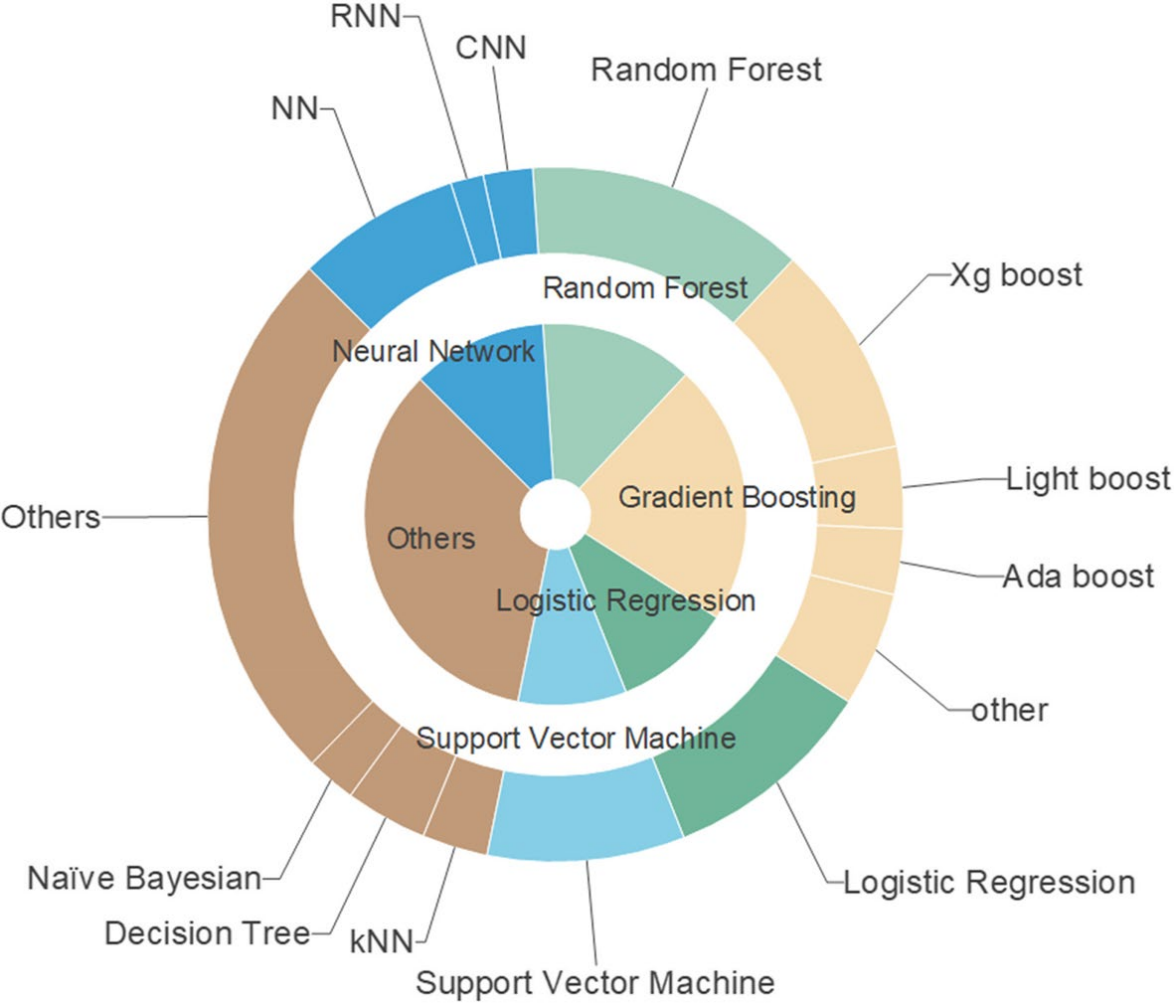
Summary of the machine learning method from studies in our system review

In summary, there are 49 different ML systems deployed. The most common algorithm is the random forest with 17 (32.7%) usages. A different variation of gradient boosting algorithms is also very common with 13 (25%) XGBoost, 4 (7.7%) adaboost, and 7 (13.5%) others. Neural networks methods and its variances were also albeit less frequent with 8 (15.4%) neural network (NN), 1 (1.9%) deep neural network (DNN), 2 (3.8%) recurrent neural network (RNN) and 3 (5.8%) convolutional neural network (CNN) for 14 (26.9%) in total. Existed ML-based models were also tested for example ALI sniffer, Dense-Ynet and ResNet-50 (Fig. 3).

The definition and phenotypes of ARDS were defined recently using the Berlin definition and updated in 2023 [76]. Therefore, there were various attempts to establish a more rigorous subphenotype using the ML algorithm over the years. Unsupervised algorithms were used with some success. Sinha [31] used latent class analysis to separate patients into hyper and hypo-inflammatory states. Zhang et al.[38] in 2019 and Liu et al. [53] in 2021 both tried to categorise ARDS patients into 3 subphenotypes using tree-based gradient boosting and k-mean method respectively. Although the ML algorithm has shown great potential to define ARDS subphenotypes, only 6 (50%) out of 12 studies in severity and subphenotype topics used this method.

**Fig. 4:**
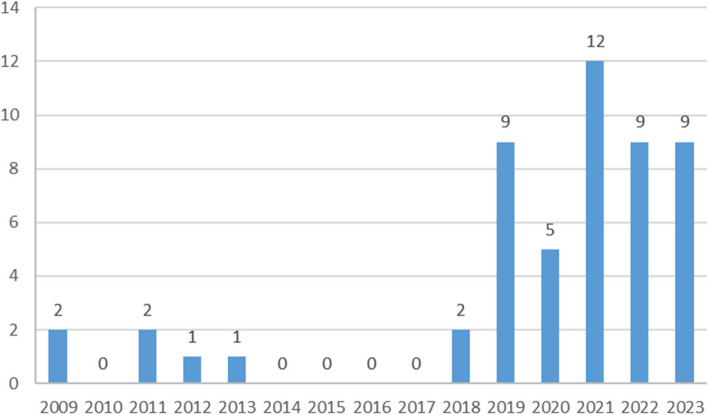
Time scale of articles on Machine learning in ARDS application

There has been a surge in ARDS research since 2019 most likely in response to the COVID-19 pandemic. 44 (84.6%) studies were published between 2019 and June 2023 of which 5 are directly used data from Covid patients (Fig. 4).

Supervised ML algorithms are widely used for many applications such as verifying subphenotypes, improving diagnoses, predicting the development of ARDS, potential outcomes and providing insights into the management of ARDS. Across these applications, the gradient boosting method and its variations proved to be very popular, being used in 24 of the studies. 12 (23.1%) articles employed multiple ML algorithms including gradient boosting-type algorithms: gradient boosting and its variations. Among those, Gradient boosting-type algorithms had the best performance in 8 studies (66.7%), for example, Yang [40], Reamaroon [57] and Lazzarini [58]. The most common supervised ML algorithm is random forest used in 17 studies, followed by logistic regression and extreme gradient boosting (XGBoost) in 13 studies.

**Fig. 5:**
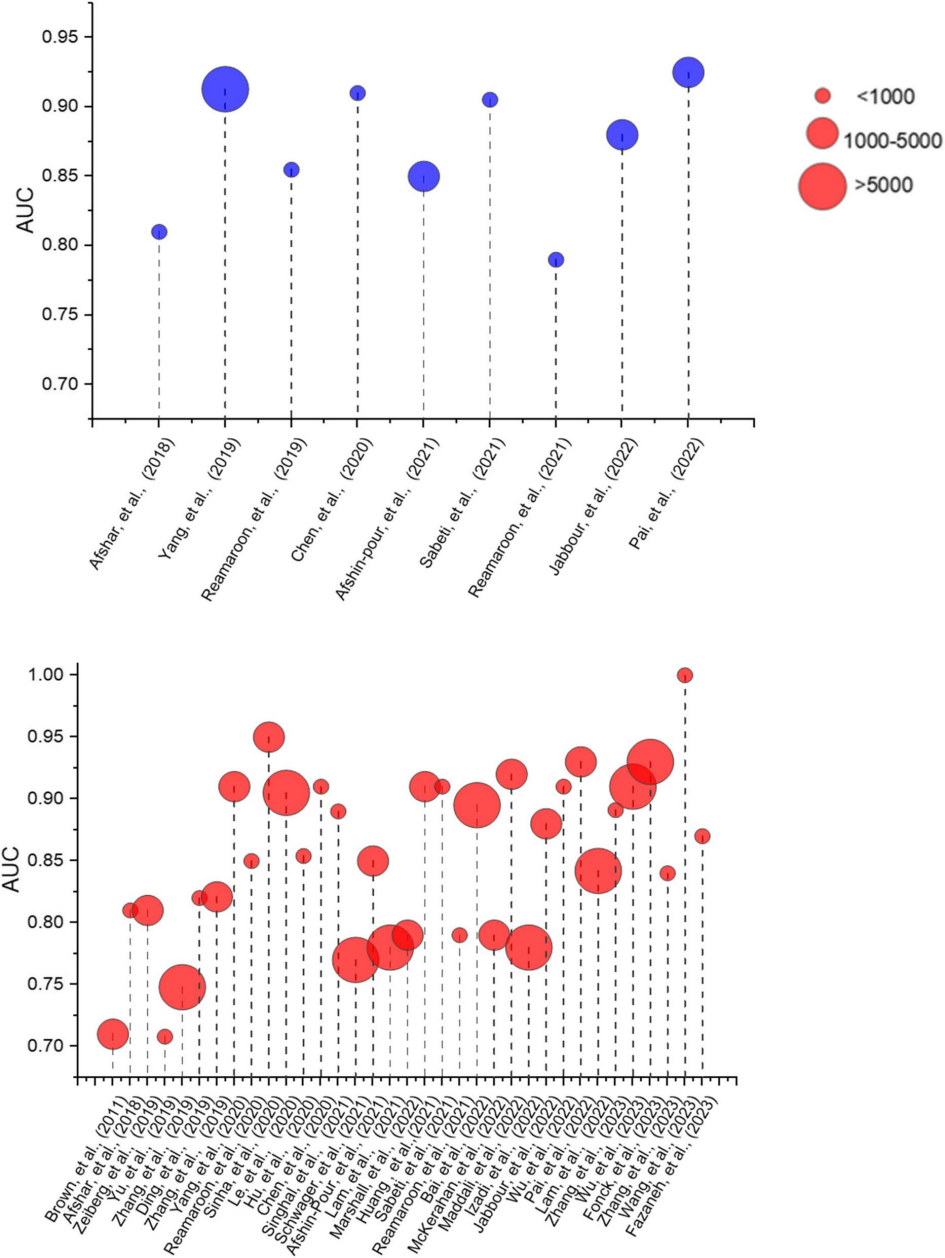
Data size and performance comparison for different ML models. Blue: Studies on ARDS diagnosis, Red: Studies on prediction of ARDS. X-axis indicates time and the size of the circles represents the size of the database used in each study

In term of data used, the most popular data source is from private data collections, which was used in 30 studies (57.7%). Public and large data collections composed the rest of data usage. The most popular public data collection is The Medical Information Mart for Intensive Care (MIMIC) and was used 12 times in two versions 3 [34] and 4 [67] (23.1%). The eICU database [51] is also popular and was used in 9 studies (17.3%). Others notable data sources include the Secure Anonymised Data Linking (SAILS) Databank [42] with 4 appearances and the National Lung, Heart, and Blood Institute ARDS Network (ARMA, ALVEOLI, and FACTT) [46] which was used 10 times across all versions. Even with large data collection like MIMIC and eICU, only 12 (23.1%) studies included more than 5000 samples (Fig. 5). The largest data collection is from the National Trauma Data Bank from the US used by Pearl, et al., [26] with 1,438,035 patients. Barakat, et al., [72] used 1 million simulated patients based on MIMIC 3 database for their study. The simulation method was developed by Sharafutdinov [77]. This approach circumvents the need of cleaning the data, data protection and deidentification and handling missing and inconsistent data. It also allows limitless database in term of data size.

In 14 studies there was an attempt to develop algorithms based on neural network architectures. The developed models based on neural network architecture such as ResNet-50 (CNN) and Dense-Ynet (DNN) were also tested with promising results such as with Jabbour in 2022 [63] and Yahyataba [71] in 2023. However, when competing with non-neural network models in Yang [40] in 2019, Izadi [62] in 2022, Xu [47] in 2021 and Wang [67] in 2023, neural networks showed no advantage in terms of ROC area under the curve (AUC) or accuracy. This might be due to the amount of data available for use in the neural network (Fig. 5), showcased clearly in Lam [66] 2022 study, developing XGBoost and RNN model on the relatively large database of 40,703 patients with RNN came out on top with AUC = 0.842.

**Fig. 6:**
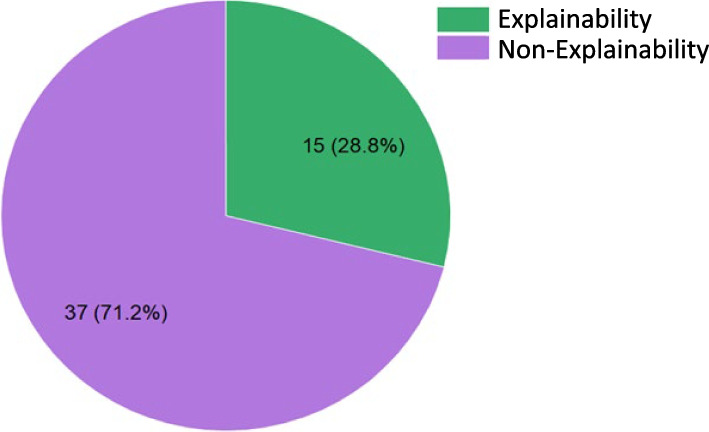
Pie chart identifies the percentage of explaination models in total reviewed articles

There are 15 (28.8%) studies which employed explainability in ML in some way (Fig. 6). The most popular explainability method was feature importance used in 13 (87%) studies. Most of these studies did not specify how the feature importances were obtained. 6 studies used feature extraction tools: Shapley additive explanations (SHAP) and Local interpretable model-agnostic explanations (LIME) to obtain the importance of all the features that contributed to the results [49, 58, 65–67, 73]. In 2020, Sinha et al. [46] used feature importance on 3 different ML methods to determine the 6 most impactful parameters which can be fed into the final ML algorithms. The white-box approach of explainability was used by Wu et al. in 2022 [69] via an interpretable random forest algorithm. Wang et al. in 2023 [68] used 3 different feature attraction methods SHAP, LIME and DALEX for their best-performing algorithm. They were also the only group that actively pursue explainability as the core feature of the final algorithm.

## Discussion

This review aimed to highlight the usage of ML methods on ARDS and ARDS-related issues such as diagnosis and management. The vast majority of research showed good results within their performing metric, for example, all studies used AUC as a performing gauge and archived the AUC values of between 0.7 and 1. However, while most studies employed the k-fold validating technique and/or used separated cohorts for validation, only one study by Lazzarini et al.[58] compared and validated the prediction capability of the ML algorithm through clinicians.

XGBoost seems to be the most popular and successful algorithm. This may be due to the size of the database used in these studies [24, 33, 40, 42, 48, 51, 57–59, 65, 66, 69, 73]. While large public databases such as MIMIC and eICU were commonly used, the vast majority of research used less than 5000 samples. This may limit the viability of more advanced ML algorithm such as neural network and its variances. Additionally, ML algorithms especially non-neural network models, can perform well with limited data, having a large database can potentially provide a more stable and reliable final algorithm. The most advanced ML algorithm, neural network, also requires a larger database to increase its potential. However, collecting patient data is meet with many difficulties in term of ethic and administrative control such as identifiability or patient consent. An interesting way to avoid this is by using virtual/simulated patients pioneered by Barakat [72]. However, whilst this method provided arbitrarily large, cleaned and complete database, the realistic of the virtual patients must be thoroughly tested and justified before being used for ML model development. It is another layer of complexity added on top of the developed ML model which must be independently validated.

With the rise of applications of ML and AI in real life, medical law, regulations, and demand for transparency will require a larger degree of explainability on ML algorithms. However, the use of explainability methods in the reviewed articles seems to be an afterthought with only one research actively trying to create an explainable ML algorithm as one of their main goals [67]. Furthermore, there was no attempt to validate those explainable features with actual physicians and clinicians. With the growing impetus and demand for digital healthcare, more research in this area is required. For example, there is currently no method to quantify the effectiveness of explainability methods to clinicians that was utilised in the included papers. Future work also should verify the resulting ML algorithm and is explainability methods with actual physician and clinician as a key component of the research. Although a rigorous validating method was proposed by Amarasinghe et al. [78], there are currently few studies that fully utilise this method [78].

To bridge the gap between research and real-life application, future research should focus on not only the performing metric of the ML algorithm such as AUC or accuracy but also on finding a clear explanation for the algorithm outcome. These should not be limited to graphical outputs such as those provided by SHAP or LIME but should other outputs (textual or numerical). Validating these explanations with clinicians and physicians should also be prioritised. We propose another validation step by seeking consensus with clinicians to validate the usability of future models.

The risk of bias was not formally reported in this review due to bias assessment tool such as Prediction model Risk of Bias Assessment Tool (PROBAST) is for prediction model alone. However, in general, the characteristic of data used such as ethnicity or sex were unreported in all studies. Therefore, the risk of bias is high in all studies if PROBAST was used.

To develop more robust ML model, there is a need for a large, multinational, multi centres database. This database will help to reduce bias, increase representation in different ethnic and gender groups. Collaboration between clinician and data scientist is also vital to cross validate and evaluate the viability of developed model. One of the most important purposes of the reviewed studies is to further the knowledge about ARDS and thus provide a tool for clinician to improve patient’s condition and survivability. Therefore, a rigorous framework for assessing the effectiveness of explainability of ML model on end-user is needed. The framework may contain series of surveys and tests to evaluate clinicians’ performances with and without ML support and explanations. Such framework would narrow the gap between academic study and real-world applications.

## Conclusion

This systematic review captures the usage of ML in ARDS research. This is the most extensive review on this topic thus far with 52 articles included. However, due to the amount of area of research included, spanning 7 categories (Fig. 2), meta-analysis was not considered for this paper. This can be done in future review focusing on each category of ML application.

Machine learning has been proven to be useful in many aspects of ARDS including diagnosis, risk assessment, mortality prediction and prognosis. To fully utilise the advantages of neural network algorithm, a database of more than 5000, ideally more than 10,000 patient records is required. With small databases of fewer than 5000 records, extreme gradient boosting has the highest probability of success. Public databases such as MIMIC are ideal if used in conjunction with handpicked data to either provide a broader spectrum, or to validate the resulting algorithm emerged from such data. With such database, more advanced and powerful ML algorithm such as neural network, reinforcement learning and deep learning and be utilised and show their full potential.

In term of area of research, not a lot of research focused on how ARDS is currently managed (Fig. 2). More research could be done in this category such as in drug admission and ventilator setting as improvement in this area can vastly improve the mortality rate of patients. As the nature of this kind of the outcome of management research is more complex than prediction of ARDS or mortality research, this category of research would also benefit from lager database and more advanced algorithm mentioned above.

In terms of explainability, while SHAP and LIME are popular choices, there is still a gap between understanding and utilising the results from such instruments by data scientists compared to real clinicians. Therefore, to develop a machine learning model to truly support clinicians to tackle ARDS, there is still a lack of research on transparent and explainable models. Due to the complexity of ARDS in definition, recognition, and management, this is challenging. Future research and studies on machine learning applications in ARDS should focus more on the explainability and robustness of the model rather than the accuracy and sensitivity of the models.

Amarasinghe et al. [78] proposed a framework to quantify the effectiveness of explainability method to clinician. This method involves a series of survey on how clinician’s opinion changed with and without explainability. Future research can ultilised this method to evaluate the resulting algorithm and explainability method. This can accelerate the acceptance and integration of ML into real life application. However, this method is time consuming due to the number of clinicians required and the number of surveys needed for this method to be statistically significant. Therefore, a more approachable framework that requires fewer resources, would be hugely beneficial for future researches and can be integrated into more researches.

## Data Availability

No datasets were generated or analysed during the current study.
